# Simulation Palynologists for Pollinosis Prevention: A Progressive Learning of Pollen Localization and Classification for Whole Slide Images

**DOI:** 10.3390/biology11121841

**Published:** 2022-12-16

**Authors:** Lin-Na Zhao, Jian-Qiang Li, Wen-Xiu Cheng, Su-Qin Liu, Zheng-Kai Gao, Xi Xu, Cai-Hua Ye, Huan-Ling You

**Affiliations:** 1 Faculty of Information Technology, Beijing University of Technology, Beijing 100124, China; 2 Beijing Meteorological Service Center, Beijing 100089, China

**Keywords:** pollinosis prevention, whole slide images, progressive learning, deep learning, pollen localization and classification

## Abstract

**Simple Summary:**

Pollen allergy is a highly prevalent disease affecting humans worldwide. Early pollen identification can help allergic individuals to prevent pollinosis. Recently, automatic pollen identification (API) has been shown to play a prominent role in pollen concentration monitoring. Developing an accurate and effective identification system may provide new insights for pollinosis prevention. This paper presents a novel automatic pollen identification method integrating localization tasks and classification tasks, thus perfectly mimicking the observation process from palynologists. The inter-task dependence and intra-task reliability are simultaneously considered in this method to effectively enhance the pollen identification performance. We believe that our study will contribute to enhancing symptom control of pollen allergy and maintaining the life quality of allergic patients.

**Abstract:**

Existing API approaches usually independently leverage detection or classification models to distinguish allergic pollens from Whole Slide Images (WSIs). However, palynologists tend to identify pollen grains in a progressive learning manner instead of the above one-stage straightforward way. They generally focus on two pivotal problems during pollen identification. (1) **Localization:** where are the pollen grains located? (2) **Classification:** which categories do these pollen grains belong to? To perfectly mimic the manual observation process of the palynologists, we propose a progressive method integrating pollen localization and classification to achieve allergic pollen identification from WSIs. Specifically, data preprocessing is first used to cut WSIs into specific patches and filter out blank background patches. Subsequently, we present the multi-scale detection model to locate coarse-grained pollen regions (targeting at “pollen localization problem”) and the multi-classifiers combination to determine the fine-grained category of allergic pollens (targeting at “pollen classification problem”). Extensive experimental results have demonstrated the feasibility and effectiveness of our proposed method.

## 1. Introduction

Allergic disease is considered a global health concern [1] and cited by the World Health Organization (WHO) as one of the three major diseases of the 21st century [2]. Pollen allergy, commonly known as hay fever [3], has become the most widespread allergic disease as the continuous advancement of urban afforestation. A recent report [4,5] indicates a general increase in the incidence of pollen allergy with a profound socio-economic impact. Approximately more than 10% of adults and 40% of children worldwide suffer from pollen allergy [4], and the annual cost for allergy management and drug therapy reaches up to billions of dollars [5]. Once allergic pollen comes into contact with the oral, nasal, or eye mucosa of allergic patients, it will cause a spectrum of clinical symptoms (e.g., allergic rhinitis, exacerbating asthma and conjunctivitis, etc.) [6]. Early identification and treatment can effectively enhance symptom control of pollen allergy and maintain the life quality of allergic patients. Therefore, it is essential to provide accurate pollen concentration monitoring information on the occurrence of airborne pollens for highly allergic individuals.

The standardized workflow for pollen concentration monitoring has been established in many countries, including twin pillars of pollen sampling and pollen identification [7,8]. In China, the Durham pollen sampler [9] has achieved significant success for pollen monitoring and the Hirst volume sampler [10] is regarded as a biomonitoring gold-standard device in Europe. The Burkhard [11] and Lanzoni samplers [12], based on the Hirst sampler, are also widely used in other countries. The procedure of pollen identification is uniform across countries, which always requires visual recognition of each pollen taxon under the microscope by well-experienced specialists. However, pollen identification is accepted as the most labor-intensive and time-consuming part of the concentration monitoring step. This process involves a fully manual observation and takes about half the time of the whole pollen monitoring process [13], which may result in a delay of relevant risk alerts and negative health effects for allergic individuals.

The automatic pollen identification (API) task aims to accelerate the process of providing up-to-date concentration information of pollen grains to allergic sufferers [14]. The API problem was first stated by Stillman and Flenley [15] more than 20 years ago. It gained a great deal of attention as soon as it was proposed in the palynology community. The API is expected to be applied in some cases to work on a practical operation, especially various computer-aided systems. The current mainstream of API studies is mainly based on Scanning Electric Microscope (SEM) [16,17,18,19] and Light Microscope (LM). SEM images usually have high resolution, which makes the pollen grains differentiate obviously. Even though this is technically feasible, the expensive cost and strict requirements for sample preparation limit its practical application [20]. By contrast, LM is more common in meteorological monitoring stations due to its simple operation, convenient actual deployment, and inexpensive cost [21,22]. Therefore, the LM-based API task has received increased attention from researchers.

Advances in computer vision techniques have further promoted the development of LM image-based API research [23]. Many scholars in this area have reached a consensus that classification and detection models can independently address API problems in a one-stage manner. The former classification model focus on categorizing all pixels in a given image into specific pollen classes based on semantic content [24,25,26,27,28,29,30,31]. One of the significant successes is the classic work [29], which extracts shape and texture descriptors and then generates Support Vector Machine (SVM)-based classifiers. These approaches heavily rely on artificial prior, making the classification process subjective. The deep learning-based models can automatically classify pollen grains without any prior knowledge [30,31]. Convolutional neural network (CNN), a core branch of a deep learning network, is widely developed and obtains impressive results [32,33,34,35,36]. For example, Sevillano et al. [30] presented three deep learning-based models, which show 97% accuracy on the POLEN23E pollen dataset. However, these classification models focus only on the category information excluding the positioning information of the objects. Besides, they are always built upon a specific assumption, namely, the image dataset only involves purified and isolated pollen grains. The real-world pollen WSIs invariably contains more complex impurities, unlike idealized experimental data. When they are directly input into the model, the underlying assumptions will not be met, leading to a serious misclassification.

Another dominant solution is to implement automatic pollen monitoring programs based on object detection models. The detection model can be seen as the generalized version of classification models. One notable property of these models is that localization and classification tasks can be integrated into the model as parallel branches (trained in an end-to-end manner). That is, the position information and category information of detected objects can be obtained simultaneously. The introduction of detection models has given birth to the rise of many breakthroughs in the API task. This method has shown excellent ability in distinguishing pollen grains from diverse and complex background features (coarse-grained: whether it is pollen). However, different allergic pollens share similar morphological structures such as shape and texture characteristics. It is extremely hard for detection models to focus on the detailed features that are highly useful for distinguishing pollen subcategories (fine-grained: which subcategory of pollen it belongs to, such as Cupressaceae, Pinaceae, etc.), which results in model over-detection and degradation of identification performance.

**Figure 1:**
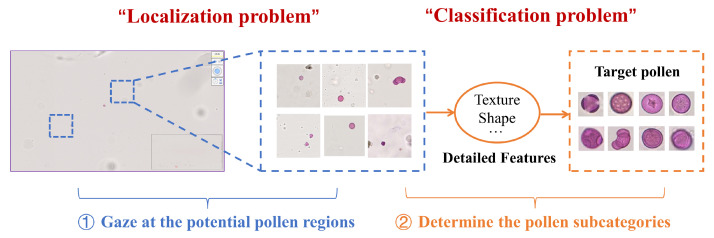
The pollen identification process in a progressive manner from the palynologists.

As a matter of fact, palynologists tend to identify the pollen grains in a progressive way instead of the above one-stage straightforward approaches. They usually focus on two pivotal problems when observing the pollen under the microscope: (i) Where are the pollen grains located? (pollen localization); (ii) Which categories do these pollen grains belong to? (pollen classification). Therefore, they generally follow a strategy of “localization first and then classification”, that is: the potential pollen regions will first be gazed at in the image; subsequently, the fine-grained categories of these pollens are determined by considering detailed features (as shown in Figure 1). The first step aims to discover all the candidate pollen grains from other complex impurities, while the goal of the second step is to match the predicted pollen with the target pollen one by one, thus assigning a specific subcategory to each pollen grain. To mimic this natural processing way of human beings, the following twofold is fully considered to design our identification model in our study:

- Inter-task dependence: From the observation process of “localization first and then classification”, we can infer that there are inherent associations between the detection and classification tasks. The localization information obtained from the detection procedure indicates potential regions of pollen grains, which is excellent guidance for the fine-grained classifiers to capture subtle discriminative patterns in specific regions. It can be expected that better identification performance will be achieved if we combine the detection with classification tasks in the computer vision community, for the information of the former task will make huge contributions to the latter task.
- intra-task reliability: Influenced by the nature of pollen slide image (e.g., complex impurity information, similar pollen features, etc.), there are some bottlenecks within the localization and classification tasks. Specifically, it is difficult to find the potential pollen regions due to diverse and complex impurities information. Besides, the instances of different allergic pollen look similar in global appearance, which is easily wrongly recognized as other subcategories. Intuitively, the reliability performance boosting of each substage helps to enhance the overall identification accuracy. Therefore, specific consideration needs to be given to how to enhance the internal reliability in detection and classification tasks.

Inspired by the above considerations, we propose a novel progressive pollen identification model by incorporating localization and classification. Different from the existing API methods that focus only on a single task, our research not only fully considers the inherent inter-task correlation to combine the location information and classification details, but also effectively enhances the intra-task identification performance of each stage by introducing the multi-scale detection and multi-classifiers combination. Specifically, data preprocessing is first adopted to cut WSIs into specific patches and filter the useless patches containing blank backgrounds. Then, we leverage a multi-scale object detection model to detect informative regions that are highly correlated with pollen from the candidate patches (coarse-grained pollen localization). Finally, each region containing pollen grain is input into a multi-classifiers combination to obtain the final pollen identification results (fine-grained pollen classification). In this way, the localization information is served as region guidance for the classification stage, making the classifiers pay more attention to subtle features of local specific regions. Not only localization information extracted from pollen detection is considered, but also fine-grained categorical information such as texture, contour, and color learned from deep learning models are exploited. The contributions of this paper can be summarized as follows:

- Considering the inter-task dependence of pollen detection and classification, we present a novel multilevel progressive learning to achieve automatic allergic pollen identification from real-world LM images. The pollen WSIs are performed by data preprocessing to filter the useless patches containing blank backgrounds. The coarse-grained localization provides the coarse position information that indicates the pollen region (targeting at “localization problem”), and the multi-classifiers combination is utilized to learn detailed discriminative features related to each pollen subcategory (targeting at “classification problem”).
- Considering the intra-task reliability of pollen detection and classification, the multi-scale and multi-classifiers feature learning methods are introduced for reinforcing pollen identification performance. The multi-scale feature fusion helps to localize pollen regions from complex impurities by enlarging the receptive field, while the multi-classifiers feature representation combines different base classifiers in a parallel manner, making the model more effective in distinguishing different allergic pollen from each other.
- Extensive experiments are conducted based on the real-world pollen dataset, which includes 2971 WSI images labeled with 8 + X categories (all other unknown pollen or debris are aggregated in an “X” category). Results of comparison experiments and ablation studies prove the effectiveness and superiority of our proposed method.

## 2. Materials and Methods

### 2.1. Datasets

Recent advancements in deep learning have accelerated many new applications for automatic palynology image analysis. One of the key drivers for the success of deep learning is the availability of large amounts of training data. Most public datasets are sourced from abroad [25,30,31,37,38,39], as shown in Table 1. The geographical area variations may result in differences in the distribution and composition of pollen species. Thus, the collection of pollen grains from specific areas is significant for pollen forecasting in those areas. To realize automatic palynological analysis for Chinese patients, an allergic pollen dataset containing native pollen WSIs (APD-WSI) is established in our study, which is strongly supported by the Beijing Meteorological Service Center (BMSC).

#### 2.1.1. Data Collection

**Figure 2:**
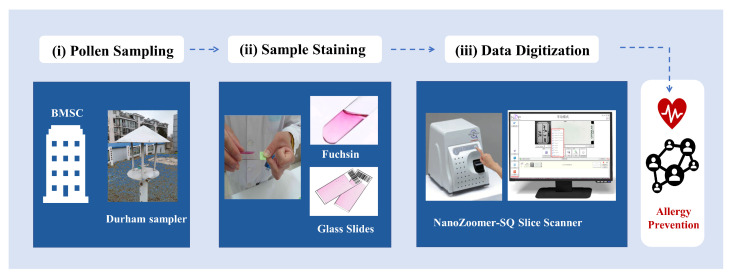
The overall acquisition processing of pollen WSIs.

Our WSIs are obtained using light microscopy which is commonly widely used at palynological monitoring stations. The overall acquisition processing of WSI mainly includes three pillars: pollen sampling, sample staining, and data digitization, as shown in Figure 2. Specifically, the staffs regularly collect pollen samples from the Durham sampler by the gravity sedimentation method. Subsequently, prepared samples are uniformly stained with an appropriate amount of Fuchsin to highlight the pollen grains placed on microscope slides. Finally, we use the NanoZoomer-SQ Slice Scanner (a specialized digital slice scanner) under an X40 magnification to digitize the entire glass slide thus generating the pollen WSIs.

**Table 1:** The list of publicly available pollen datasets (all other unknown pollens are aggregated in an “X” category).

Dataset	Region	Year	Pollen Types	Grains	Image Types	Resolution
Duller ’s Pollen Dataset [37]	Unknown	1999	7	630	Grayscale	25 × 25
POLEN23E [25]	Brazilian Savannah	2016	23	805	Color	Varying (Minimum 250 pixel per dimension)
Pollen73S [31]	Brazilian Savannah	2020	73	2523	Color	Varying (Average size 512 × 512)
New Zealand pollen [18]	New Zealand and the Pacific	2020	46	19,500	Color	227 × 227
Pollen13K [38]	Unknown	2020	4 + Debris	>12,000 + ∼1000 examples of debris	Color	84 × 84
Cretan Pollen Dataset v1 [39]	Crete	2021	20	4034	Color	Varying
**Ours**	Beijing, China	2022	8 + Debris	10,080 + X	Color	110,000 × 50,000

#### 2.1.2. Professional Labelling

The WSI not only can completely reproduce the real morphology of pollen particles on glass slides, but also have outstanding color fidelity compared with the glass slide. The significant characteristics of WSI are summarized as threefold: (1) The imbalance of foreground and background in WSI biases observation towards useless background rather than foreground objects; (2) a large number of complex impurities (such as dust, insect debris, plant debris, etc.) are mixed with the pollen grains; (3) the pollen subcategories share similar visual features.

**Figure 3:**
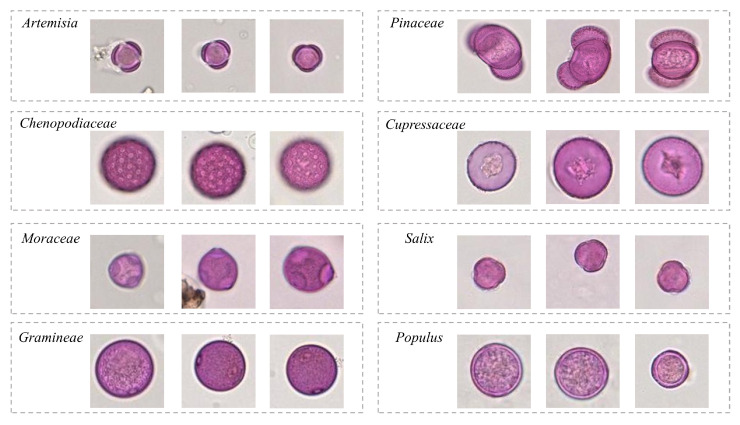
Some examples of each allergic pollen class.

To adapt these properties of WSI, we follow the professional palynologists’ recommendation and divide the labeling process of each WSI into three steps:

1. Foreground labelling. All original WSIs are firstly preprocessed into numerous patches based on the non-overlapping cutting strategy described in Section 2.3 Subsequently, the patches without any microscopy object (e.g., pollen, impurities, bubbles, etc.) are labeled as blank background by the experts and the others are considered as ground truth labels of the foreground images.
2. Coarse-grained region annotation. Even though the patches with foreground objects can be selected through the above step, the position information of pollen grains in each patch has not been known. Therefore, the palynologists are invited to label the pollen regions, impurity areas, and bubble regions in selected patches with bounding box-level annotation. The pollen grains usually exhibit a regular shape and are pink-like in color. In terms of the impurities, they have special-shaped objects with yellow or brown color, while the bubbles normally present colorless and circular shapes. Our goal is to effectively distinguish coarse-grained pollen regions from non-pollen noise interference in this way.
3. Fine-grained subcategories labelling. Based on the above region-level annotation, the palynologists further label allergic pollen categories for each region. A total of eight pollen species are listed as main inhalation allergens in Beijing, include Artemisia, Gramineae, Chenopodiaceae, Cupressaceae, Pinaceae, Populus, Salix, and Moraeeae, respectively, since these taxa cover with 90% of the total amount of pollen in Beijing area. Out of these eight taxa, Cupressaceae, Salix, Populus, and Pinaceae are known to be prevalent in spring, while Moraceae, Compositae, and Chenopodiaceae are the main pollen contributors in autumn. All the above-mentioned eight allergic pollen are labeled in our dataset by the staff. Some examples of each pollen class are represented in Figure 3.

#### 2.1.3. Dataset Statistics

**Figure 4:**
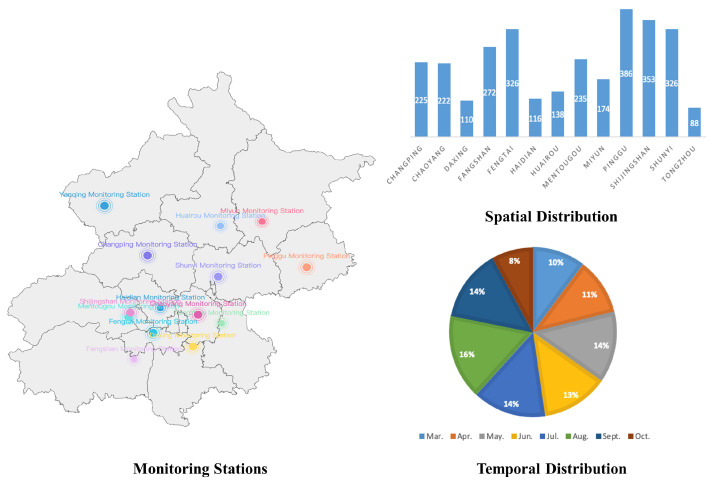
Statistics of monitoring stations, spatial distribution, and temporal distribution in our dataset.

The APD-WSI dataset includes a total of 2971 WSIs, each of which with gigapixels in size (up to 50,000 × 110,000 pixels). Considering that pollen dispersal differs largely across regions and periods, we choose 13 pollen monitoring stations in Beijing, China as primary study plots and continuously collect pollen samples from March 1st to November 29th annually (other months are non-flowering stages). Figure 4 shows the map of the Beijing region and the location of airborne pollen monitoring stations. The histogram of the spatial and temporal distribution of the APD-WSI dataset is also shown.

### 2.2. Methods

**Figure 5:**
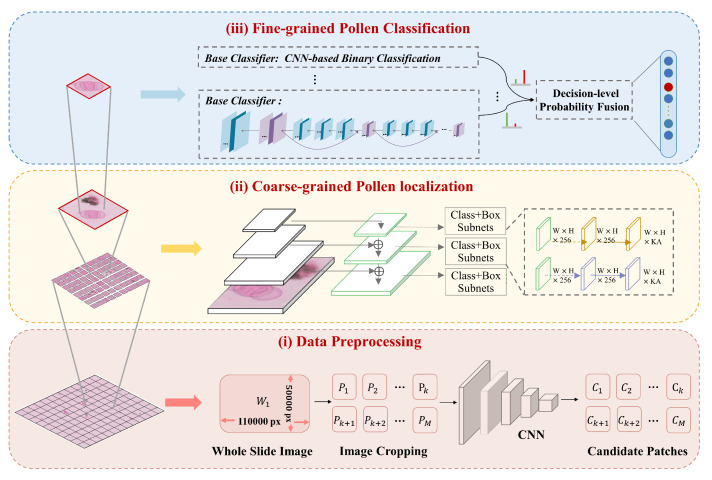
The overall network architecture of our proposed method.

In this section, we introduce the details of the progressive allergic pollen identification model integrating localization and classification from WSIs. Figure 5 shows the overall framework of the proposed method. Our proposed method contains three parts: (1) **Data preprocess:** the image preprocessing is employed to cut the WSI into fixed patches and remove blank background patches; (2) **Coarse-grained pollen localization:** we propose a pollen detection network to locate pollen grains considering multi-scale image features; (3) **Fine-grained pollen classification:** the multi-classifiers combination is presented to achieve fine-grained allergic pollen classification.

### 2.3. Data Preprocessing

The original pollen WSI must be preprocessed due to its gigapixel size and extremely imbalanced foreground-background. We first cut pollen WSIs into numerous images with small sizes called patches. This design draws on a variety of recent ideas from [40,41] and leverages OpenSlide [42] and NDPItools [43]. The *W* and *P* notation is defined to distinguish between “pollen WSIs” and “image patches” that correspond to that image. Given an image *W* with gigapixel resolution L*H (*L* refers to length, *H* refers to width. *L* ≥ 110,000, *H* ≥ 50,000) at 40× magnification, it is cut into a number of non-overlapping *P* with size of l*l. Considering that the WSIs are taken from pollen slide samples in a real scene, the position distribution of pollen grains is basically random inside the image. To adapt these properties and comprehensively retain image information, we followed the professional palynologists’ recommendation to cut each WSI into non-overlapping patches. This manner provides a broad view field for detection and classification programs, enabling most of the pollen grains observed. All cropped patches form an image sequence with the length of *M*. In our experiments, *l* is set to 512 which fits the input shape of the subsequent step, and here M is more than 20,000 (variable due to the total area of pollen WSIs). Subsequently, we train a CNN-based binary classifier to classify each patch as one of the foreground classes or as the background. Each patch is passed to the CNN model, and a probability vector is calculated to predict the label of each patch via the Softmax function. The confidence threshold is set to 0.5. The patches are assigned to the foreground label when their classification probabilities exceed 0.5, and to the background label if their confidences are in [0, 0.5). When training the model using our dataset, the results of Alexnet can pursue the best classification performance, which is much better than other classical classifiers. In this way, we filter out the majority of irrelevant patches with a blank background while retaining the informative patches containing the foreground objects. The above outcome is used to generate the candidate patch set to serve as input for the next phase. The *C* is used to denote the candidate patches. It is noted that solely due to the introduction of the combination of non-overlapping cutting and a CNN-based patch filter, M rapidly narrows down to a small number (e.g., 1–2 k), which effectively reduces the computational burden of the subsequent module.

### 2.4. Coarse-Grained Pollen Localization

The detection module is responsible for locating the potential regions of pollen and removing complex impurities in the candidate patches. Rather than most detection models that only focus on a single feature map from the last layer of the network, we extract and fuse multiple feature maps with different receptive fields of the given input image. The RetinaNet [44], a classical single-shot detector [45], is used as our basic detection model (as shown in Figure 5). Noted that this section solely detects the presence of pollen grains, and the categorization happens in the independent next step.

**Basic Multi-Scale Detection.** The RetinaNet basically involves 4 dedicated deep convolutional neural networks. The ResNet50 [46] is first employed as a backbone network to extract convolutional features. Then, the Feature Pyramid Network (FPN) [47] is combined with the backbone network for integrating multiple scale features. The FPN constructs a pyramid hierarchy to aggregate image features from low level to high level, which is implemented through the top-down pathway and lateral connections. Finally, two task-specific subnets are simultaneously performed in a parallel manner. One is for assigning anchor boxes to pollen classes and another one is for regressing from anchor boxes to ground-truth object boxes. The Focal loss (FL), a variant of a CE function, is used as a loss function to penalize the classification subnet, which is responsible for forcing the CNNs to focus on training instances that it finds most difficult to classify (as shown in Equations (1)).

(1) $$FL\left(p_{t}\right)=-{\left(1-p_{t}\right)}^{\gamma }\log\left(p_{t}\right)$$

For the regression subnet, the Smooth L1 loss is introduced to calculate the regression loss by the offset of the coordinate. We define the bounding box information of the ground truth as $V=\left(v_{x},v_{y},v_{w},v_{h}\right)$ and the location information of the predicted anchor box as $T=\left(t_{x},t_{y},t_{w},t_{h}\right)$. Accordingly, the regression $L_{loc}$ loss can be expressed as:

(2) $$L_{loc}=\sum_{i\;\in \;(x,y,w,h)}^{}Smooth_{L_{1}}\left(t_{i}-v_{i}\right)$$

in which,

(3) $$Smooth_{L_{1}}\left(x\right)=\left\{\begin{array}{cc} x^{2} & \;\mathrm{if}\;\left|x\right|=1\\ \left|x\right|-0.5 & \;\mathrm{otherwise}\; \end{array}\right.$$

**Localization guidance.** The multi-scale feature enhancement during the detection procedure makes localization performance more accurate, effectively mitigating the irrelevant interference of impurity areas. Besides, the potential region proposals obtained from the pollen detection task can basically be viewed as effective guidance information about the existence or non-existence of pollen grains. The region guidance can guide the model to allocate more attention to pollen regions with key discriminative features. By embedding pollen detectors before classifiers (the next stage), the inherent relations between pollen localization and classification can be fully considered, which is helpful for greatly improving pollen identification accuracy.

### 2.5. Fine-Grained Pollen Classification

After detecting the regions containing pollen grains, we further design a novel classification model for determining final fine-grained classification results. In our practice, single multi-class classification networks are directly used to address pollen subcategories identification. However, they fail to obtain satisfactory distinguishing results, which attributes to the fact that some similar features shared between pollen subcategories inevitably confound the model, resulting in disempowering the discriminative ability of the model for each fine-grained class. Inspired by the ensemble learning mechanism [48,49], we transform multi-class classification problems into multiple binary classification problems. In this way, the multi-classifiers combination is proposed to boost the performance of fine-grained pollen identification.

The multi-classifiers combination comprises a set of customized standard binary classification networks. Encouraged by professional palynologists, they generally match the visual features of predicted pollen with target pollen one by one when encountering a similar pollen identification problem. Based on this practical experience, the number of base classifiers of ensemble combination is set to the number of allergic pollen labels (here the number of labels is eight). The OVR-based (One vs. Rest) training mechanism is used to prepare the training data of each base classifier. Specifically, we divide the c-class dataset into c subsets to train specific binary sub-classifiers, where the *k*th subset is composed of positive instances with the *k*th class and negative ones with rest classes. On this basis, each base classifier relies on individual training data.

With regards to the selection of an independent base classifier, the CNN-empowered networks are perceived as the backbone model to learn different class-specific discriminative features. The commonly used CNN models include AlexNet [27], ResNet [46], VGGNet [50], and DenseNet [51], etc. It has been verified that deep learning methods outperform traditional classification models driven by manual feature engineering. The feature representation ability of different CNN-based classifiers is not constant across different pollen types, as evidenced in the experimental results in Section 3. The ideal combination of base classifiers on the best integration performance is further explored. Accordingly, we adopt the specific-class CNN classifiers with the highest performance metric to construct an optimal combination of multiple learning paths.

The decision-level probability fusion is finally designed to fuse different basic classifiers as a unified probability representation. Our fusion strategy takes the maximum probability of multiple independent models to determine the ultimate class label, each targeting a specific pollen class. The maximum method decides the results according to the most reliable classifier. Noted that we do not use the weighted operation to ensemble multiple classifiers, as the balanced weight is fair for all pollen categories. In the situation where some of the classifiers will not be chosen, our fusion model is still capable of making predictions. For example, when the sampling date of pollen slides is known, only the classifiers corresponding to pollen classes that are likely to occur in the current periods and regions need to be selected. This step makes the multiple base classifiers complement each other and improves the fine-grained classification accuracy.

## 3. Results

In this section, we carry out extensive experiments to evaluate our proposed approach. The separate experiments are set up in a three-stage manner: background classification, coarse-grained pollen localization, and fine-grained subcategories classification. Besides, the overall identification performance was also estimated, which further reveals the prediction capability of our proposed method.

### 3.1. Experiment Settings

**Figure 6:**
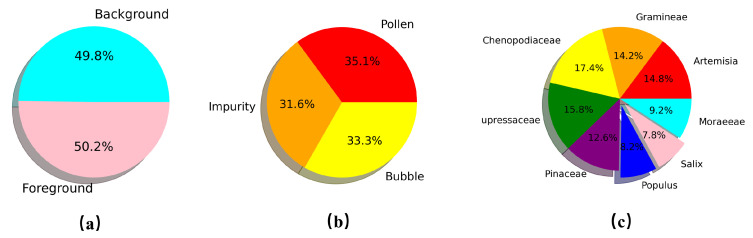
The data distribution of (**a**) D1, (**b**) D2 and (**c**) D3 datasets.

We construct three image subsets based on our APD-WSI dataset for training and testing each stage of the proposed model(they are named D1, D2, and D3), which are labeled by professional palynologists. Specifically, the first D1 dataset consists of 18,000 background images and 18,175 foreground images. This dataset is used to train and test CNN which is responsible for filtering background patches, where each image is labeled with a binary value of 1 or 0. The 10,324 impurities, 10,893 bubbles, and 11,500 pollen images are contained in the D2 dataset. These data are served for a coarse-grained detection model with the class labels and coordinates positions of all ground truth bounding boxes. The D3 dataset includes 10,080 images covering eight pollen subcategories, which is considered the training and testing data of the fine-grained classification task. The detailed information of the three datasets is shown in Table 2. Figure 6 exhibits the data distribution of D1, D2, and D3 datasets. In terms of the D3 dataset, the numbers of the Populus label and Sailx label are imbalanced with other categories. Such data augmentation operations are employed to balance data sampling, including random vertical flip, horizontal flip, and 90-degree rotation. The ten-fold cross-validation is applied in this paper for evaluation purposes.

To speed up model convergence, weight training on ImageNet is used as weight initialization in our experiments. For parameter training, the Stochastic Gradient Descent (SGD) was selected as the optimizer with a batch size of 16. The initial learning rate and momentum are set to 0.001 and 0.9, respectively. At each iteration, the loss of the model is recorded to show the variations observed during the model training. The maximal number of iterations is set to 100, which is the default value. The training is conducted until the validation loss no longer decreases between consecutive training cycles. Our proposed approach is implemented using the PyTorch framework.

The data preprocessing is responsible for cropping the image into patches and allowing the distinction of background and foreground objects by modeling a novel CNN. The different patch sizes (256, 512, and 1024 pixels) are used to train the proposed CNN model. The accuracies of the background classification model are 90.94%,94.82%, 89.52% with respect to patch sizes of 256, 512, and 1024 pixels. When the patch size equals 512, we obtain the best distinguishing ability. Accordingly, the patch size is chosen as 512 to generate patch sequences for subsequent pollen identification.

**Table 2:** The detailed description of three sub-datasets for training and testing our proposed model.

Dataset	Aim	Data Distribution (Class-Number)	Size
D1	Data preprocessing	Background-18,000/Foreground-18,175	512 × 512
D2	Coarse-grained pollen localization	Pollen-11,500/Impurity-10,324/Bubble-10,893	512 × 512
D3	Fine-grained pollen classification	Artemisia-1640/Gramineae-1580/Chenopodiac-1930/Cupressaceae-1750/Pinaceae-1400/Populus-910/Sailx-870/Moraeeae-1020	100 × 100

### 3.2. Evaluation on Coarse-Grained Pollen Localization

**Figure 7:**
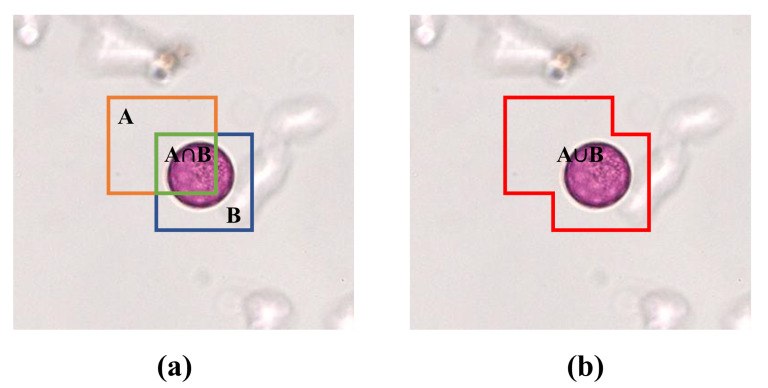
(**a**,**b**) The graph interpretation of IoU. A refers to the predicted bounding box and B refers to the ground-truth bounding box.

The coarse-grained pollen localization described in Section 2.4 can locate individual pollen in each image patch. Different from general detection models, the multi-scale feature is additionally considered to enhance the discriminative ability of pollen grains from complex backgrounds. In this section, we compare our multi-scale detection model with other state-of-the-art detectors. The Average Precision (AP) is measured to evaluate the detection performance in our experiment. The AP is the average precision over Intersection over Union (IoU) from 0.5 to 0.95 evaluated at steps of 0.05. The IoU is the ratio, ranging from 0 to 1, of the overlapping area of the ground truth and predicted areas to the union area. Figure 7 shows the graph interpretation of IoU and its formula is seen as Equation (4). Additionally, the AP50 and AP75 are adopted in our experiments, which represent the average precision when the threshold is 0.5 or 0.75.

(4) $$IoU=\frac{area(A)\cap area(B)}{area(A)\cup area(B)}$$

Table 3 shows the comparison of the detection performance between our multi-scale detector and other state-of-the-art detection models, including Fast RCNN [52], Faster RCNN [53], YOLO family [54] and SSD [55]. For a fair comparison, the other models are retrained over our D2 training set. As we can see in the experimental results, our proposed detection model has greater values of the AP, AP50, and AP75 compared with the other detection algorithm. Especially, the AP of our model is 0.034 higher than other best methods, which demonstrates the effectiveness of our approach. We additionally evaluate the impact of the multi-scale feature enhancement mechanism on pollen detection. To this end, we simply remove the feature pyramid construction, and then investigate the pollen localization results with and without the multi-scale feature fusion. The results are shown in Table 4. As shown, the detection performance can be significantly enhanced by introducing multi-scale feature fusion. In contrast, the AP value will decrease once the multi-scale is not incorporated. Thus, the above results verify the necessity of feature pyramid construction for pollen localization in our approach.

### 3.3. Evaluation on Fine-Grained Pollen Classification

The fine-grained pollen classification aims to solve the problem of allergic pollen identification. Rather than current classification models, our module integrates multiple classifiers to empower the perception ability between different pollen subcategories. The classification performance is qualified by accuracy metric.

We first investigate the impact of different CNN-empowered classifiers on the classification performance of various pollen subcategories. The state-of-the-art classification networks represented by Alexnet, Vgg-16, ResNet-50, and DenseNet-121 are selected as our compared models in this experiment. Each network is designed as a binary classification task, i.e., whether the specific type of pollen grain exists or not. Here, we consider eight pollen subcategories as our main experimental targets, which is consistent with the dataset described earlier. The accuracy results of each model on different subcategories are shown in Table 5. As we expected, different classifiers have varying abilities to identify various pollen subcategories. Specifically, the DenseNet achieves the best performance on Cupressaceae and Populus classification with an accuracy of 91.80% and 80.22%, yet it performs worse than the ResNet when identifying Chenopodiaceae and Sailx. Moreover, the optimal performance is obtained by leveraging VGG-16 on predicting Artemisia, Graminea, and Moraeea with 87.50%, 85.10%, and 72.10% accuracy, respectively. Regrettably, the AlexNet fails to show superior performance on across-category classification. These findings have implications for the designing strategy of multi-classifiers combination. Inherently, the choice of combining multiple classifiers usually is either homogeneous (e.g., using the same type of classification subcomponents) or heterogeneous (e.g., using different types of classification subcomponents). Due to variability in classification accuracies of different CNN-empower classifiers on specific classes, the heterogeneous structure is ultimately applied in our multi-classifiers combination to improve predictive performance.

**Figure 8:**
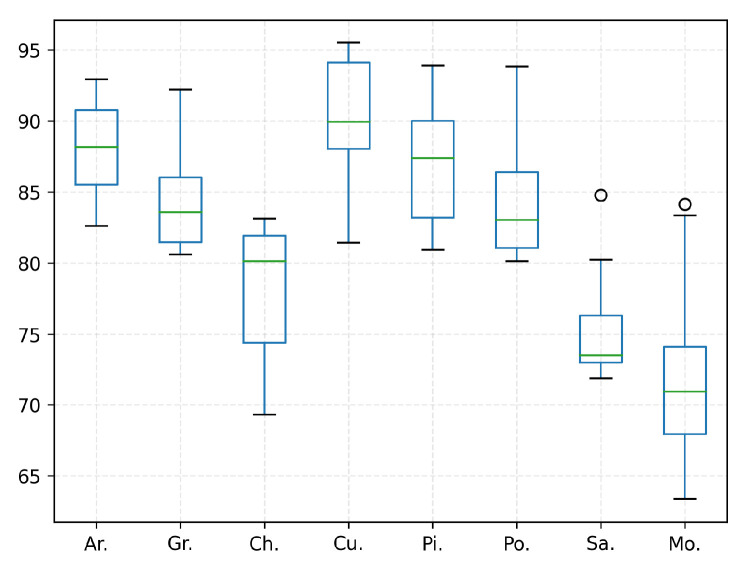
The boxplot of the dispersion of ten-fold cross-validation experimental results across different pollen categories.

We further explore the identification performance of our proposed multi-classifier combination. The standard classification models are used as the representative of the base classifiers in this section. Unlike the above-mentioned validation trial in Table 5, one prominent experiment design is that the classification task is extended to multi-class classification. This contributes to assuring a fair comparison in our experiment. Both multi-classifiers combination and single multi-class classifiers are responsible for assigning the input image to one of the main pollen subclasses of interest to us (eight items are studied in our experiment). In detail, Table 6 presents the results of comparative analyses, and Figure 8 shows the distribution of ten-fold cross-validation experimental results of our proposed model. It is apparent that our multi-classifiers combination obtains significant identification performance than the other models across all categories.

### 3.4. Evaluation on Overall Identification Performance

In the previous subsection, we have demonstrated that intra-task reliability can be improved by multi-scale feature enhancement and multi-classifiers integration. Further detailed experiments are performed in this section to validate the superiority of leveraging inter-task dependence. In our approach, the final prediction results are generated by progressively progressing three different stages, i.e., data preprocessing, coarse-grained pollen localization, and fine-grained pollen classification. Additionally, we add a direct sampling that only involves cutting operation without patch filtering to compare with our data preprocessing method.

**Figure 9:**
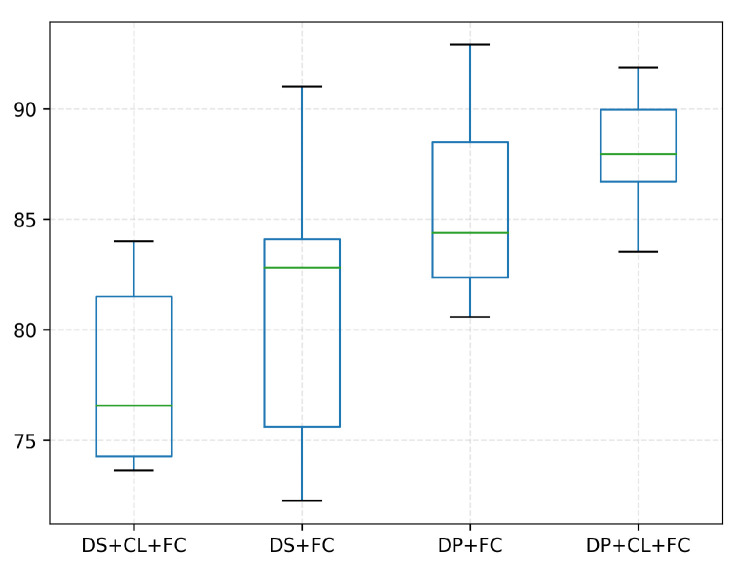
The boxplot of the dispersion of ten-fold cross-validation ablation experimental results. The short form is used to facilitate reading: DS refers to Direct Sampling, DP refers to Data Preprocessing, CL refers to Coarse-grained Localization, FC refers to Fine-grained Classification.

**Figure 10:**
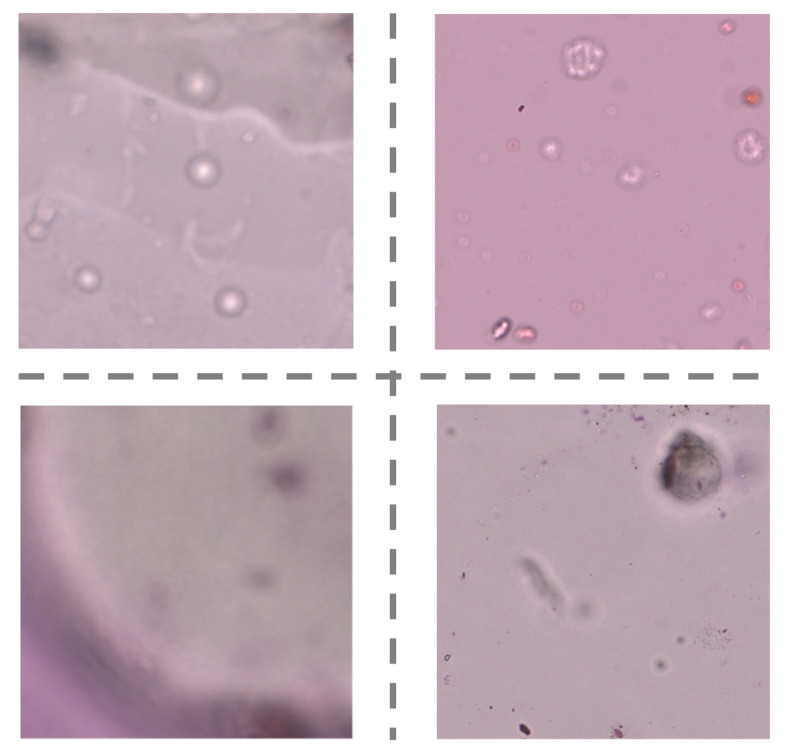
Some examples of misclassification from “Direct sampling+ Fined-grained classification” combination.

**Figure 11:**
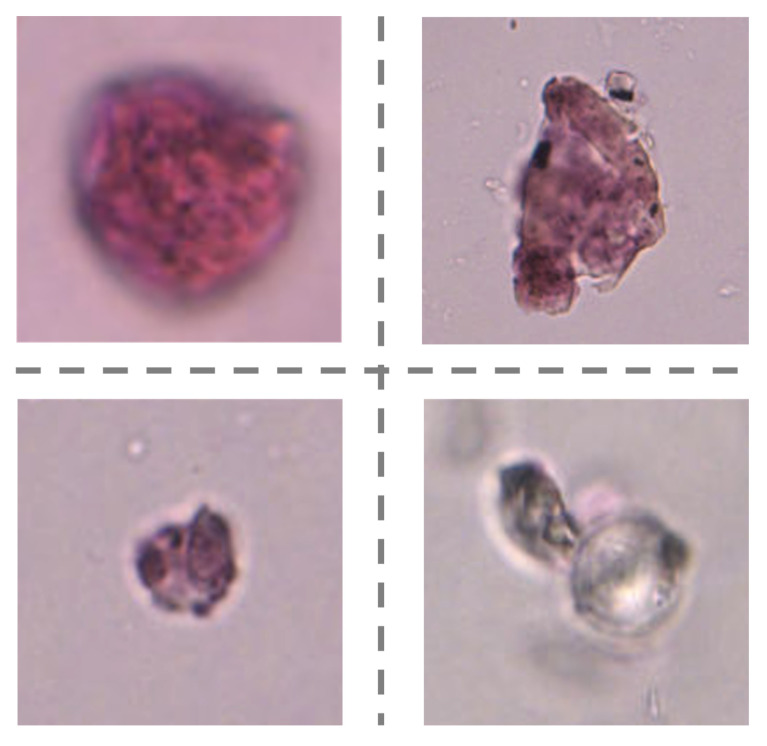
Some examples of misclassification from “Data preprocessing+ Fined-grained classification” combination.

Table 7 shows the classification performance from four cases, effectively expressing the inter-task dependence. The dispersion of ten-fold cross-validation ablation experimental results of our model are shown in Figure 9. We can observe that the accuracy of “Data preprocessing+ Coarse-grained localization+ Fined-grained classification” is higher than that of “Direct sampling+ Coarse-grained localization+ Fined-grained classification”. It has proved that the introduction of patch filtering contributes to improving identification accuracy. This view is also verified in the accuracy comparison between “Data preprocessing+ Fined-grained classification” and the “Direct sampling+ Fined-grained classification” combination. As shown in Figure 10, the examples are all mistaken for pollen in “Direct sampling+ Fined-grained classification”, but it can be correctly detected as background and successfully filtered out by the patches filtering in “Data preprocessing+ Fined-grained classification”. Given the experiment results, the reinforcing relation between image preprocessing and the subsequent tasks are obviously shown: the pretreatment for the original image could effectively minimize the noise interference from complex background. It is of great significance for overall performance enhancement. The guidance relationship between the localization task and classification task is explored as well. As we have seen in Table 7, the “Data preprocessing+ Coarse-grained localization+ Fined-grained classification” combination boosts the categorization accuracy significantly, which brings 2.5% improvements compared with “Data preprocessing+ Fined-grained classification”. We found that “Data preprocessing+ Fined-grained classification” fails to correctly identify the samples of Figure 11 as impurity class, but the “Data preprocessing+ Coarse-grained localization+ Fined-grained classification” combination can accurately detect them. It is not difficult to speculate that the localization information from the detection task can be served as region guidance for the classification stage. This makes the classifiers obtain better performance by focusing on subtle features of local specific regions.

## 4. Discussion

### 4.1. Model Performance

Through extensive experiments, we can see that our proposed progressive identification framework provides a better solution to classify fine-grained allergic pollen. It performs better than some approaches only based on single detection or classification task. The accuracy of a single detection-driven or classification-driven identification model on our dataset is much lower than the results obtained by our proposed method. This may be due to the fact that the above research typically treats two tasks independently, without considering the inter-task dependence between them. In fact, the localization information obtained by the detection task can make the classification task easier to focus on specific regions that can be inferred as pollen labels. This is of great significance to resist the interference of impurity information and ensure the accuracy of pollen detection.

The superior performance of our proposed method is also shown in inside localization and classification tasks. The tasks themselves proved to be challenging tasks due to the inherent features of pollen data (e.g., complex impurity information, similar pollen features, etc.). Despite the state-of-the-art detection models or classification models used in our experiments, they did not work well in our designed tasks. As we can see from Table 3, the SSD detector achieved the best performance on our dataset compared with any other basic models, but actually only obtained an average accuracy of 0.772. Different from the above-mentioned basic models, we introduce multi-scale feature fusion in our coarse-grained pollen localization, which prompts the model to take into account the multiple features of pollen images. The effect of multi-scale feature fusion on detection performance has been experimentally demonstrated in Table 4. The 0.816 AP, 0.925 AP50, and 0.847 AP75 are finally yielded through our multi-scale localization model, which clearly outperforms all other detection approaches.

Table 6 exhibits the significant advantages of our proposed multi-classifiers combination in classification performance. Specifically, our algorithm achieves optimal identification accuracy across different categories. This mainly benefits from integrating multiple classifiers in a parallel manner. In our ensemble combination, the high-performance classifier is chosen as the base classifier. The combination of the multiple optimal base classifiers provides the basis for considerably improving the predictive power of the overall classification model.

### 4.2. Practical Implication

Our proposed approach provides a basis for establishing a deep allergic pollen forecasting service in the meteorological and palynological community. The performance of allergic pollen identification is an extremely urgent task. The airborne pollen is the main outdoor allergen for early spring pollinosis, which seriously affects the quality of life for people susceptible to allergies. Therefore, our progressive pollen identification framework helps allergic patients to assess the risk of disease and enhance the preventive capacity of allergic diseases in the general public. Moreover, the pollen WSIs data used in our study can exhibit the overall vegetation characteristics and the individual features of specific plant taxa within the pollen source area in Beijing, China. This authenticity of pollen information helps the automatic classification system participate more accurately in identifying efforts for allergic pollen and improves the service reliability in practice.

### 4.3. Limitation and Future Work

One limitation of this study is that we only focus exclusively on eight allergic pollen in Beijing, China. In the future, more types of samples collected from wider geographic regions are needed to increase the wide utility of the model in practice. Additionally, we acknowledge that class imbalance hinders the equal representation of different data types, which results in poor generalization ability of the deep learning algorithms. A larger sample size is required to balance the data distribution between different pollen classes, thus further increasing the identification power of the model. In future work, we will adopt data augmentation methods and transfer learning to make up for them.

## 5. Conclusions

In this paper, we develop a progressive learning model integrating pollen localization and classification to address the “localization problem” and “classification problem”. The model considers the inter-task dependence and intra-task reliability to perfectly mimic the manual observation process of the palynologists: The data preprocessing module is firstly designed to cut each WSI into patches with specific sizes and filter useless patches. Then we present the multi-scale pollen detection model to localize coarse-grained regions of pollen grains from candidate patches (to solve the “localization problem”). Finally, the multi-classifiers combination is proposed to determine the fine-grained categories of allergic pollens (to solve the “classification problem”). We demonstrate through extensive experiments that our proposed method can work reliably with high accuracy on real-world pollen data. However, our method only studies a relatively small number of pollen species and suffers from the class imbalance problem, which is a serious constraint for greater generalization and application. We hope to address these issues using data augmentation and transfer learning methods in the future.

## Figures and Tables

**Table 3:** The comparison results of AP between our proposed detector with the other state-of-the-art detection models.

Model	AP	AP50	AP75
Fast RCNN	0.635	0.728	0.664
Faster RCNN	0.782	0.868	0.849
YoloV3	0.535	0.725	0.604
YoloV5	0.586	0.792	0.649
SSD	0.772	0.895	0.821
Ours	0.816	0.925	0.847

**Table 4:** The independent ablation experiments to verify the effectiveness of multi-scale feature fusion.

Method	AP	AP50	AP75
W/ Multi-scale Feature Fusion	0.816	0.925	0.847
W/O Multi-scale Feature Fusion	0.729	0.830	0.781

**Table 5:** The accuracy results of different classifiers on various pollen subcategories.

Subcategories	Alexnet	Vgg-16	ResNet-50	DenseNet-121
Artemisia	58.13%	87.50%	82.93%	74.70%
Gramineae	52.90%	85.10%	77.63%	59.06%
Chenopodiaceae	60.27%	64.93%	70.93%	62.43%
Cupressaceae	64.63%	84.76%	71.33%	91.80%
Pinaceae	65.71%	81.07%	85.35%	78.57%
Populus	66.53%	79.90%	77.30%	80.22%
Sailx	53.96%	56.59%	67.58%	52.19%
Moraeeae	51.93%	72.10%	63.27%	55.37%

**Table 6:** The comparison results between single multi-class classifier and multi-classifiers combination (Species abbreviations are composed of the first two letters from each type of allergic pollen).

Models		Ar.	Gr.	Ch.	Cu.	Pi.	Po.	Sa.	Mo.
Single Multi-class Classifier	AlexNet	55.12%	58.12%	63.11%	71.14%	67.14%	66.59%	55.40%	53.14%
	Vgg-16	84.76%	83.22%	68.13%	86.29%	83.57%	78.57%	57.47%	70.10%
	ResNet-50	83.23%	79.11%	72.02%	74.29%	84.29%	76.92%	70.11%	67.75%
	DenseNet-121	79.27%	72.15%	61.13%	80.57%	77.14%	81.32%	54.02%	57.35%
Multi-classifiers Combination	Ours	88.11%	84.49%	78.24%	90.29%	87.14%	84.09%	75.29%	72.20%

**Table 7:** The performance of components in our progressive identification approach.

Direct Sampling	Data Preprocessing	Coarse-Grained Localization	Fine-Grained Classification	Accuracy
✓		✓	✓	77.82%
✓			✓	80.50%
	✓		✓	85.70%
	✓	✓	✓	88.20%

## Data Availability

The data that support the findings of this study are available from the corresponding author, upon reasonable request.
